# Clinical features of invasive fungal disease in children with no underlying disease

**DOI:** 10.1038/s41598-021-03099-w

**Published:** 2022-01-07

**Authors:** Juan Huang, Chentao Liu, Xiangrong Zheng

**Affiliations:** 1 Department of Pediatrics, Xiangya Hospital, Central South University, Xiangya Road, Changsha City, 410008 Hunan Province People’s Republic of China; 2 Department of Pediatric, The First Hospital of Changsha, Changsha, Hunan China

**Keywords:** Outcomes research, Paediatric research

## Abstract

There is limited research into Invasive fungal disease (IFD) in children with no underlying disease. We undertook a retrospective study of children with IFD who did not suffer from another underlying disease, from June 2010 to March 2018 in Changsha, China. Nine children were identified. Eosinophil counts were elevated in six cases. The level of procalcitonin (PCT) was elevated in six cases. Fungal culture was positive in all patients, including eight cases of Cryptococcus neoformans and one case of Candida parapsilosis. 8.33 days following antifungal treatment, the body temperature of the eight patients affected by cryptococcal disease had returned to normal. Our study indicates that the primary pathogen in IFD was Cryptococcus neoformans in children who had no other underlying disease. Eosinophils can be considered to be indicators of cryptococcal infection. IFD in children with no other underlying disease has a satisfactory prognosis.

## Introduction

Invasive fungal disease (IFD) has emerged as an important cause of human disease over the past three decades. It often involves deep tissues and blood, or even damaged organ tissues, which distinguishes it from superficial mycosis^1^. IFD is also known as a severe, deep, disseminated, and systemic fungal infection^2^. Many factors have contributed to the emergence of IFD, including the HIV epidemic, organ transplantation, chronic disorders such as chronic lung diseases, immunosuppressive therapies, and the increase in the number of frequent hospitalizations and interventions. It has also gradually emerged in the immunocompetent population. Studies in different regions have shown that the mean incidence of IFD ranges between 14.1 and 27.2 cases per 100,000^2–4^, and is associated with both high morbidity and mortality^5^. More than 2,000,000 people are affected by life-threatening fungal infections every year, creating a tremendous global burden of disease^6^. *Candida* has always been the most common pathogen in IFD, followed by *Dimorphic Fungi*, *Aspergillus* and *Cryptococcus*. The entire body is susceptible to fungal infection, including the brain, lung, bone marrow, liver, and spleen. A diagnosis of IFD is assisted by reference to the international consensus guidelines^7^. However, there was limited space in the guideline document for some population groups, including pediatrics, neonates and immunocompetent people. In this study, we collected the clinical data from IFD children with no other underlying disease, between June 2010 and March 2018 in Changsha, China. The aims of this paper are to provide more data on IFD in children, and to increase pediatrician awareness of IFD, especially in children with no other underlying disease. A literature search uncovered no similar reported research.

## Materials and methods

### Patients

The medical records of all children diagnosed with "Invasive Fungal Disease" were collected from the pediatric ward of Xiangya Hospital, Central South University, between June 2010 and March 2018. Nine cases of IFD with no other underlying disease were considered.

### Data

Data extracted from the medical records included history, clinical presentation, C-reactive protein (CRP), procalcitonin (PCT), and fungal pathogen characterization. Detailed treatment plans and effects were also collected. We were given free access to the patient information. The follow-up period for all patients was 1 year.

### Diagnostic criteria

Diagnostic criteria were according to the Revised Definitions of Invasive Fungal Disease from the EORTC/MSG Consensus Group (2008)^7^. Inclusion criteria were: (1) histopathological, cytopathological, or direct microscopic evidence of fungi in biopsy specimens which were from a sterile site; (2) recovery of a mold or “black yeast”, or yeast by culture (i.e., blood, bone marrow, cerebrospinal fluid, deep lymph nodes) obtained using a sterile procedure from a normally sterile site. Exclusion criteria were: (1) underlying disease, such as a blood disease or malignancy (including receiving hematopoietic stem cell transplantation); (2) use of immunosuppressive drugs; (3) abnormal immune function (including cell immune function and T lymphocyte immune function); (4) other chronic diseases (such as nephrotic syndrome, diabetes); (5) newborns.

### Ethics statement

This is a retrospective study which only collects the clinical data of the patients, and the treatment plan of the patients is not interfered, so there is no physiological risk to the patients. The study was reviewed and approved by the Ethics Committee of Xiangya Hospital (No. 202112240). The authors confirm all data has informed consent obtained from parents/legal guardians of the children involved in the study. All methods were carried out in accordance with relevant guidelines and regulations.

## Results

Overall, 18 patients fulfilled the criteria for IFD. Among these, eight had underlying diseases including acute leukemia (7/18) and immunodeficiency disease (1/18). Nine children were included in the final analysis.

### General observations

IFD was more frequent in males (seven cases) than females (two cases). The median age was 5.98 ± 3.13 (range 2.75–13) years. One patient had a confirmed history of contact with domestic pigeons. Another had a history of exposure to old dust. The remaining had no specific risk factor history. All patients took antibiotics prior to diagnosis. The median time from onset to diagnosis was 34 ± 10.76 (range 25–57) days. Detailed clinical data and primary clinical characteristics are given in Tables 1 and 2 respectively.

**Table 1:** The detailed clinical data of 9 patients with IFD.

ID	Gender	Age(year)	Clinical presentation	The peak of fever (℃)	E (× 10^6^/L)	CRP (mg/l)	PCT (ng/ml)	BG/GM	Culture			Biopsy	Treatment			The time of fever after treatment (℃)
									Blood	Marrow	CSF		Affected organs	Before diagnosis	After diagnosis	
1	M	5.33	Fever, skin damage	39	18.8	73.6	0.2	–	CN	CN	–	CN	Lung, marrow, liver, spleen, skin	Sulfoxil	Amphotericin B Liposome	9
2	M	6	Fever, cervical lymphadenopathy	39	1.8	76.9	0.2	–	–	–	–	CN	Brain, lymph node	N	Amphotericin B Liposome, Fluorocytosine	2
3	M	4	Fever, cough	39.7	7.2	257	1.05	–	–	–	–	CN	Lung, liver, spleen, lymph node	Meropenem, Vancomycin	Amphotericin B Liposome, Fluorocytosine	5
4	M	8	Fever, cough	39	1.6	57.5	0.072	–	–	–	CN	N	Lung, brain, liver, spleen	Sulfamethoxazole Trimethoprim, Azithromycin	Amphotericin B Liposome, Fluorocytosine	12
5	M	3.5	Fever, bellyache, skin damage	40	0.4	70.3	0.59	–	–	–	CN	CN	Lung, brain, skin	Vancomycin, Teicoplanin	Amphotericin B Liposome	11
6	M	7	Fever	40	5	63.3	0.33	–	–	–	CN	CN	Lung, brain, liver, lymph node	Piperacillin Tazobactam	Amphotericin B, Fluorocytosine	13
7	F	4.25	Fever, bellyache	40	14.7	108	0.71	–	CN	CN	CN	CN	Lung, brain, marrow, lymph node	Ceftriaxone, Cefoperazone Sulbactam	Amphotericin B Liposome	14
8	M	2.75	Fever	41	0.4	54.4	3.43	–	–	CN	CN	CN	Lung, brain, marrow, liver, spleen, lymph node	Aztreonam	Amphotericin B, Fluorocytosine	8
9	F	13	Fever, limb weakness, vomiting	39	0.2	52.4	8.34	156/40.16	CP	CP	–	N	Lung, marrow	Ceftriaxone	Give up	

*CRP* C-reactive protein, *PCT* procalcitonin, *BG* (1–3)-β-D-glucan (BG), *GM* galactomannan, *CSF* cerebrospinal fluid, *CN* *Cryptococcus neoformans*, *CP* *Candida parapsilosis*,—negative, *N* n.

**Table 2:** The primary clinical characteristics of 9 patients with IFD.

Variable	Number of patients (%); mean (± SD)
**Demographics**	
Age(years)	5.98 (± 3.13)
Male	7 (77.8%)
**Symptom**	
Fever	9 (100%)
Fever peak (℃)	39.63 (± 0.7)
Bellyache	2
Skin damage	2
Cough	2
Vomiting	1
Limb weakness	1
Eosinophil count(× 10^9/L)	8.18 (± 7.08)
CRP (mg/L)	90.37 (± 64.71)
PCT (ng/ml)	2.82 (± 3.29)
Positive BG/GM test	1
**Fungi**	
Cryptococcus neoformans	8 (89%)
Candida parapsilosis	1 (11%)
The time required for the diagnosis (days)	34 (± 10.76)
The time required for fever relief (days)^a^	9.25 (± 4.13)

^a^Just for the children infected by *Cryptococcus neoformans.*

### Clinical presentations

All patients had a fever. In two of these cases, fever was the only presenting symptom. Furthermore, severe headache associated with unremitting fevers existed in these patients, one for 9 days, one for 20 days and the other for 50 days. The median peak temperature was 39.63 (± 0.7) °C (range 39–41 °C). Fever type for all children was irregular. In addition, one child had right-sided limb weakness, one presented with vomiting, two presented with skin damage, two had abdominal pain, and two had a cough. Seven had hepatosplenomegaly and enlarged lymph nodes (e.g., mediastinal, hilum, portal, and retroperitoneal).

### Laboratory and microbiological examination

**Figure 1:**
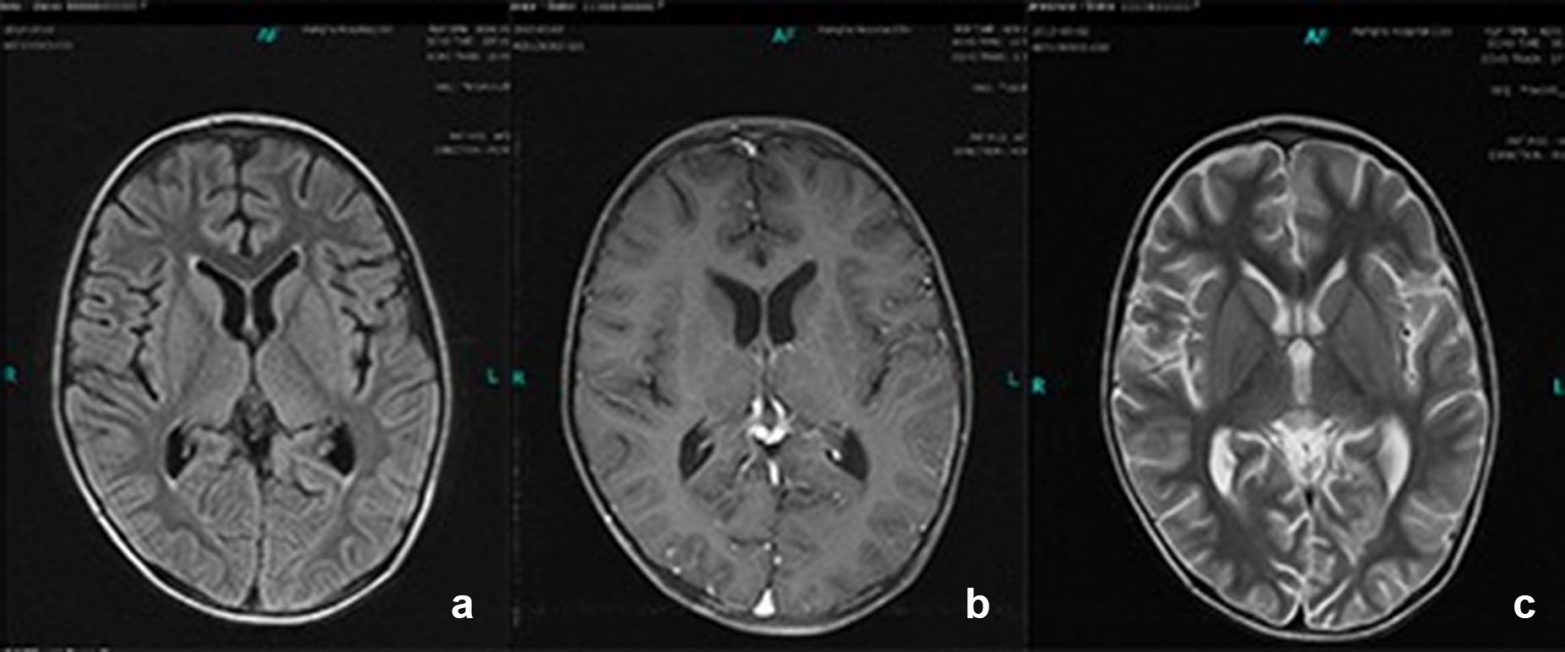
Head magnetic resonance imaging of case 5. Multiple spotted signals were seen in the bilateral white matter of the ventricle, and obvious enhancement was observed. The ventricular system was dilated.

**Figure 2:**
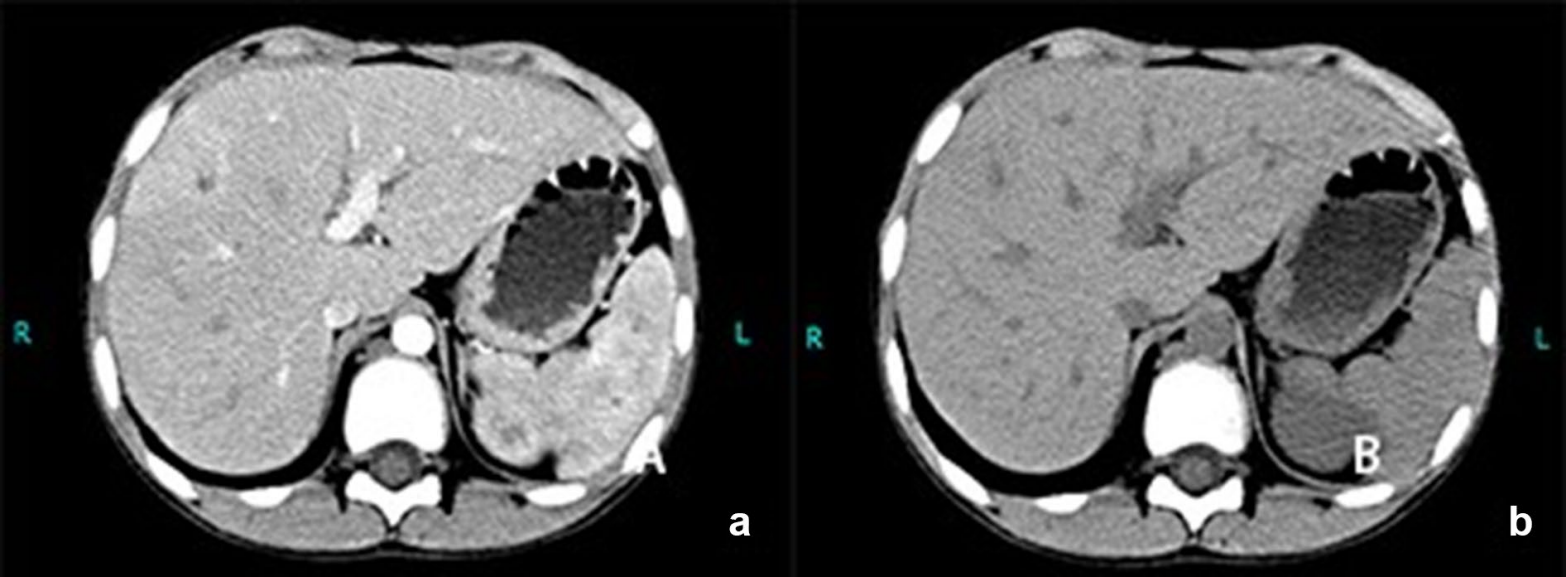
Abdominal imaging of case 4. Multiple flaky low-density lesions in the liver and spleen, and multiple enlarged lymph nodes in the hilar region, mesentery, and retroperitoneum.

Six cases had elevated peripheral blood eosinophil levels in the range of (1.6–18.8) × 10^9^ /L. CRP levels (90.37 ± 64.71 mg/L, range 24.5–257) were elevated in all patients. PCT levels were greater than 0.5 ng/ml in six cases, with a median PCT of 2.82 (± 3.29) ng/ml (range 0.59–8.34). The (1–3)-β-D-glucan (BG) and galactomannan (GM)BG and GM tests were performed in nine patients, with one being positive. Cell immune function and T lymphocyte immune function were normal in all patients. Fungi were cultured in the biopsy specimens (including blood, bone marrow, cerebrospinal fluid, lymph node and skin) of all patients. Significantly, bacterial culture was negative in all patients*. Cryptococcus neoformans* were isolated in eight cases, *Candida parapsilosis* in one. The most common infected organ was the lung (8), followed by the brain (6) (Fig. 1), liver (5) (Fig. 2), lymph nodes (5), marrow (4), spleen (4) and skin (2). The evidence of infection comes from imaging, culture and biopsy.

### Treatment and outcome

One child infected with *Candida parapsilosis* refused treatment after diagnosis. The remaining patients underwent successful antifungal treatment. Eight patients infected with *Cryptococcus neoformans* received intravenous antifungal therapy as soon as the diagnosis of IFD was established. This treatment decision was made according to guidelines published by the Infectious Diseases Society of America in 2010^8^. Average time to fever remission following inception of antifungal therapy was 8.33 (± 4.8) days (range 5–21). It was remarkably noted, when the fungal infection complicating CNS diseases, the treatment process should be monitored whether existed side effect. Actually, during their hospitalization, all patients were applied antifungal therapy with amphotericin B liposome and fluorocytosine, or additionally provided with neurotrophic drug including ganglioside needle, mouse nerve growth factor to protect CNS. Furthermore, for patients with muscle weakness, potassium supplementary was given. During the administration of amphotericin B liposome, the antifungal effect was remarkable, but renal injury needed to be prevented. One of patients represented high levels of uric acid after treatment with amphotericin B liposome 40 mg/d for 1 month, then it was replaced with fluconazole 200 mg/d, but this also led to gastrointestinal reactions, then we stopped it and applied fluconazole capsules orally for discharge. Taken together, all these patients continue received antifungal therapy with fluconazole orally at least 1 months. At the one-year follow-up, the peripheral blood eosinophil count in six of the patients had returned to normal. The eight cases remained under control and no one had relapsed.

## Discussion

The global burden of fungal diseases is enormous. The damage caused by IFD can be extensive. Fungi are universally distributed in our surrounding environment, including in the soil and air, and especially in warm and humid climates. These fungi can cause opportunistic infections, whether in immunocompromised people or immunocompetent ones, and are associated with high mortality and high financial cost^9,10^. Identifying IFD where there is no underlying disease can be difficult, because of the non-specific clinical signs and symptoms. An isolated fever is usually the only characteristic symptom^11^. However, IFD can be accompanied by other uncharacteristic symptoms, such as skin lesions, coughing, vomiting, abdominal pain, fatigue and headache. The main clinical sites for IFD are the lung, central nervous system, liver, spleen, lymph nodes, bone marrow and skin. The lung and central nervous system are the most commonly infected organs^11^. The symptoms and signs of infection of the central nervous system in patients with normal immune function were less severe than those in patients with immunosuppression^12,13^.

Elevated eosinophil counts usually occur with allergic diseases and parasitic infections. But only 67% of patients infected with *Cryptococcus neoformans* in our study had abnormal eosinophil counts. No similar finding was identified in the one case of *Candida parapsil*osis. Several in vitro and in vivo experiments suggest that eosinophils are involved in the presentation of Cryptococcal antigens^14,15^. When *Cryptococcus* enters the human body, it binds to the Fcγ receptors and activates the T- cell immune response, leading to the accumulation of eosinophils^16^. This process is related to the capsular composition on the cell wall of *Cryptococcus*. It is not clear whether the condition can be replicated in other fungi. We speculated that elevated eosinophil levels could be used as a predictor of an infection of *Cryptococcus neoformans*.

Clinicians usually look for biomarkers to help identify the details of an infection, such as CRP, PCT, (1–3)-β-D-glucan (BG) and galactomannan (GM). CRP and PCT are important indicators of bacterial infection, however, their roles in fungal disease are controversial^17^. One study shows, for fungal infections, a combination of CRP 100–300 mg/L and PCT < 0.5 μg/L offers positive predictive values of 73%, and negative predictive values of 89% in immunocompromised patients^18^. Another study reported that low CRP and PCT < 0.5 µg/l at the onset of fever may help to distinguish fungal from bacterial infections^19^. Studies looking into the value of CRP and PCT in immunocompetent patients with fungal infections are scarce. In our study, all the cases had medium-to-high CRP levels, and more than half the cases had a moderate PCT value. The obviously anomalous CRP value may relate to the immune response caused by fungi. Many of the children had been sick for a long time before coming to the hospital, and may therefore have been accompanied by a bacterial infection at the time of admission. This phenomenon may also lead to the excessive CRP and PCT levels, but we discovered the bacterial cultures represented negative results, so we did not apply antibacterial treatment. (1–3)-β-D-glucan (BG) and galactomannan (GM) are important components of the fungal cell wall. Testing for GM in serum is recommended in patients at risk for invasive aspergillosis, and a BG test is recommended for invasive candidiasis and aspergillosis^7,20,21^. Other investigators have different opinions regarding the value of BG and GM testing^22–24^. False positives may result from the influence of various factors, including protein products, and some drugs. In our study, BG and GM tests in all the children infected with *Cryptococcus neoformans* were negative. This may be explained by the low content of BG and GM in the cell wall of *Cryptococcus neoform*ans.

Tissue culture and biopsy remain the gold standard for diagnosing IFD. To our knowledge, the most common pathogens of IFD are *Cryptococcus*, *Candida*, *Aspergillus*, and *Pneumocystis*, accounting for more than 90% of reported infections^6^. In our study, *Cryptococcus neoformans* was the most common pathogen, accounting for 89% of the cases. We therefore speculate that immunocompetent children are more susceptible to *Cryptococcus neoformans*. Some studies support this hypothesis. They surmise that this unique phenomenon may be attributed to multiple polymorphisms in the genes encoding the Fc-gamma receptor 2B (FCGR2B) in the Han population of China^25^. Further study is needed.

IFD is often associated with high mortality. For children, overall, in-hospital mortality rates of 15.8% were seen for invasive Candida infection^26^. In our study, the outcomes of IFD in children without any underlying disease, were ideal. This may be attributable to the patient's own immune status.

Several limitations affect our study: We did not determine the total number of inpatients during the study period. Especially, 5 of the 8 patients with cryptococcosis had positive culture findings from the CSF, and we just concerned what kind of infection it was, but ignored the detailed parameters of CSF. Maybe we could explore what is the distinction between fungal infections with no underlying disease and other types of infections in terms of detailed parameters of CSF. In addition, the number of cases of IFD with no other underlying disease were too limited, and this study exists geographical limitation. All these children are from China, so the clinical features of IFD in this study may not be applicable to other populations.

## Conclusions

IFD has no typical performance and can affect various organs (mainly lungs and brain) in children who have no underlying disease. It is extremely dangerous, with a wide range of invasion, and may lead to irreversible organ damage. In patients with unremitting fever and hepatosplenomegaly, fungal disease should be considered. In addition, evaluation of the condition of all organs in the body is necessary. *Cryptococcus neoformans* is considered to be the main pathogen. While elevated eosinophil counts may support the diagnosis of IFD. A clinical diagnosis of fungal diseases takes both time and money. We hope that the findings of our study help clinicians identify cryptococcal infections early, and so initiate a complete fluid culture or biopsy as soon as possible to shorten the time of diagnosis. The efficacy and outcome of IFD children with no other underlying disease tends to be good.
